# *“You have to go through it and have your children”*: reproductive experiences among women with vulvodynia

**DOI:** 10.1186/s12884-015-0544-x

**Published:** 2015-05-15

**Authors:** Nora S. Johnson, Eileen M. Harwood, Ruby H.N. Nguyen

**Affiliations:** Division of Epidemiology & Community Health, School of Public Health, University of Minnesota, Minneapolis, USA

**Keywords:** Vulvodynia, Vulvar pain, Pregnancy, Postpartum, Delivery, Qualitative

## Abstract

**Background:**

Vulvodynia is a potentially debilitating chronic pain condition affecting the vulva (external genitalia) in women, with typical age of onset during the early-to mid-reproductive years. Yet, virtually nothing is known about the thoughts, feelings and experience of vulvodynia patients regarding conception, pregnancy and delivery; including the effect that a hallmark symptom, dyspareunia (painful sex), can have on a couple’s physical and emotional ability to conceive. We sought to describe these experiences and beliefs among women with vulvodynia who were pregnant or who recently had delivered a child.

**Methods:**

The study used in-depth, qualitative exploratory interview methods to gain a deeper understanding of these experiences for 18 women with vulvar pain who were recruited from an existing, nationally-sampled prospective pregnancy cohort study.

**Results:**

Four major themes were reported by our participants. Women described their reaction to pain as volatile at first, and, over time, more self-controlled, regardless of medical treatment; once the volatility became more stable and overall severity lessened, many women began planning for pregnancy. Techniques described by women to cope with pain around pregnancy included pain minimization, planning pregnancy-safe treatment and timing intercourse around ovulation. Regardless of the degree to which interaction with health care providers before pregnancy were positive, most participants developed mistrustful attitudes toward future dealings with health care systems and providers. Nearly all women described anxiety regarding how pregnancy may change pain symptoms, yet described being hopeful.

**Conclusions:**

Women described strategies regarding reproduction such as finding a personally acceptable level of pain before planning pregnancy, and a resilience that allowed them to achieve their reproductive goals despite pain and perceived lack of assistance from healthcare providers. Therefore, future research should assess the benefits of increased psychosocial support from partners and professionals who may assist in improving resilience.

## Background

Vulvodynia is a potentially debilitating chronic pain condition occurring in the absence of any clinically identifiable disorder or disease affecting the vulva (female external genitalia). This condition affects roughly 16-18 % of the general population of women at some point in life [1], and spans across age and ethnic groups [2]. Women can be affected with vulvodynia at virtually any age and life stage; however, young women in their early reproductive years (teens and early 20′s) are at greatest risk of developing vulvodynia [3].

Vulvar pain is often classified as either provoked or unprovoked vulvodynia. Provoked vulvodynia is characterized as being brought on by a specific event (e.g. during penetrative intercourse or tampon insertion) while unprovoked does not appear to have any concrete trigger. Vulvar pain level and characteristics can differ in severity throughout the life course of the disorder and also from woman to woman [4]. Dyspareunia (i.e. painful sex) is a hallmark symptom of vulvodynia and has significant implications for a woman’s, and couple’s, physical and emotional ability to have intercourse enabling conception of a child.

Obtaining a diagnosis of the disorder can often be a complicated and laborious process. In a study conducted by Buchan et al., many women report a complicated and problematic road to diagnosis, which includes a large range of providers before obtaining the proper diagnosis [5]; while other studies found that the mean number of providers seen prior to diagnosis at greater than three [6]. With regard to seeking medical care, several reports consistently found that roughly half of women who met diagnostic criteria for vulvodynia did not seek treatment for their vulvar pain [7, 8]. Reed et al. found that only 2 % of the women who reported symptoms consistent with vulvodynia were given a proper diagnosis by a provider [2].

Once a diagnosis is finally given, medical treatment is based heavily on trial and error with studies mostly showing mixed success rates, further suggesting a complex etiology that could be different from woman to woman [9]. A typical treatment course usually starts with recommended life-style changes that reduce exposures to the vulva. Drug-based treatments could include: topical steroid or estrogen creams, topical lidocaine gel, local botox injections antidepressants, or gabapentin. Other treatment plans can also include: biofeedback, physical therapy, exercise of the pelvic floor muscles, cognitive behavioral therapy, or in extreme cases of recalcitrant localized vulvar pain, surgery (e.g. vestibulectomy) [10, 11]. Most patients typically go through an extensive combination of these treatments with only limited success [12]. When a woman does find a successful treatment course that relieves symptoms somewhat or completely, she may be instructed to discontinue the regimen because its use may be contraindicated during pregnancy or may contain unknown teratogenic properties [13].

We have conducted a systematic review and have found that virtually nothing is known about how vulvodynia may affect thoughts and feelings regarding reproduction. This formative study sought to increase our collective knowledge and awareness regarding the pregnancy-related experiences of women with vulvodynia in an attempt to highlight potential gaps that, if successfully addressed, could directly and positively impact women’s lives. Ultimately, gaining a better understanding of the relationship between vulvodynia and the experiences of reproductive planning, pregnancy, and delivery will allow development of more effective health care support for affected women and couples.

## Methods

### Conceptual framework

Consistent with other qualitative studies, there was no specific hypothesis to test, as this would be too restrictive to the exploratory nature of the research and potentially lead to biased results [14]. Instead, basic areas of interest were explored as study questions. We sought both pregnant and postpartum women to get a full picture of thoughts, feelings, and concerns about pregnancy and delivery experiences. Study questions were derived from and selected based on limited previous research [15] that had been done on the subject.

Our interview strategy guided participants through a logical flow of events from contemplating pregnancy to pregnancy and delivery and we sought to better understand if and how the experience of chronic pain remained stable, diminished, changed linearly, or in cycles. Therefore, we organized our findings using a sequence of events framework, acknowledging without specific reference, the psychosocial reactions that easily fall into Fennell’s model of four-phases of chronic illness [16]. These *a priori* interview questions were crafted to explore how vulvodynia affects the following topics before, during, and after pregnancy: 1) clinician relationships and roles; 2) partner relationships and roles; 3) control over pain and treatment regimen decision-making; 4) concerns and expectations regarding pregnancy; and 5) motherhood.

### Human subjects approval and consent

The project was approved by the University of Minnesota Institutional Review Board prior to initiation of the study. Each participant provided informed consent prior to initiating the study.

### Sample selection and study design

This study made use of convenience sampling, a common technique for qualitative research [14], in this case drawing participants from a 2010 to 2012 national online, prospective cohort study that investigated vulvar pain across pregnancy and into the postpartum period (VPREG). Women were selected for that study based on the following criteria: 1) English-speaking woman of reproductive age, 18 to 45 years old; 2) having a diagnosis or symptoms of vulvodynia prior to pregnancy or had a medical history of burning or pain on contact on the vulva for three months or longer; and 3) being currently in their first, or early trimester of pregnancy (less than 15 weeks gestation) or having given birth in the last 6 to 12 months.

We did not restrict participants to only those who had received a diagnosis because roughly half of women with symptoms do not seek care [7], therefore restricting eligibility would not have fully encapsulated our population of interest. Instead, we used a validated survey instrument; studies have shown that self-reported symptoms of burning and/or pain on contact of the vulva for 3 months or longer accurately predict a diagnosis of vulvodynia [17].

At the end of VPREG participation, women were asked to indicate their interest in hearing about future studies on vulvodynia. Seventy four women from that pool were invited by email to participate in this qualitative study. Respondents were directed to an online survey for further screening, i.e., age and specific stage of pregnancy or motherhood. Women were excluded from participation if they had used gestational carriers for the index pregnancy because we were examining the lived experience of vulvar pain during conception and pregnancy (so participants needed to have experienced conception and pregnancy), or if they did not currently live in the U.S. Once deemed eligible to participate, consent forms were emailed to participants for their review and electronic signatures. Once a completed consent form was received, the participant was contacted to complete the telephone interview, which lasted 20–45 min depending on the participant.

### Data collection procedures

Of 28 individuals who completed screening, 22 were eligible to complete the interview. Of those 22, a total of 18 individuals were interviewed by a trained and experienced interviewer (NJ). The remaining four eligible individuals either refused at the time of informed consent or were not successfully contacted for an interview. All interviews were digitally recorded after gaining authorization in writing, and verbally during the interview, and then professionally transcribed for analyses. Recruitment and interviews were concluded when theme saturation was reached, meaning there would be no new themes or information about themes obtained by continuing interviews.

### Analytic method

All transcripts were read at least twice by the lead author who discussed the development of coding themes with co-authors throughout the analysis process. Text units were coded at the level of paragraphs, sentences, or short sections of dialog using ATLAS.ti 6 (Berlin, Germany). While we sought in our analyses process themes drawn directly from participant narratives, we compared those themes to patterns of psychological adjustments that closely, but not exactly, fit a four-phase model of psychosocial experiences to chronic fatigue syndrome (CFS) outlined by Jason et al. using the Fennell Phases [16]. Similar to vulvodynia, CFS is another chronic pain condition of unknown origin. The validated Fennell Phase Inventory was used for CFS functioning and adaptation treatment purposes, and measured phases that were labeled as crisis, stabilization, resolution, and integration.

Through this extensive iterative process, themes were crystalized and solidified for reporting. All three co-authors participated in this process. Quantitative information obtained through the eligibility questionnaire (i.e. number of previous pregnancies, years since diagnosis, age etc.) was analyzed for descriptive statistics using SPSS v.20.0 (Armonk, NY).

## Results and discussion

Initially, we had assumed that vulvar pain would somehow significantly affect women’s child-bearing decisions. For example, if they were on a treatment regimen that was contraindicated during pregnancy, it was assumed that this transition would be difficult (overall, women reported the pain having little effect on their decision to have a baby; while most of the women interviewed were not taking any of the contraindicated medications, in fact most were not taking anything at all and those that did have to discontinue medication in anticipation of pregnancy had few problems with doing so). We had also assumed that many of these women would have engaged in assistive reproductive technologies or alternative methods for insemination because of the vulvodynia symptoms (most had not, those that had used ART had done so for fertility reasons not associated with their vulvodynia symptoms). In addition, we assumed most, if not all, of the participants would have had experiences with healthcare providers during the diagnosis and treatment process or had a difficult time obtaining a diagnosis (but not all had). Finally, we assumed that partner roles during pregnancy would be similar to those found in other studies covering this topic [5, 12].

### Characteristics of the women

Race and education were merged into this study from the original VPREG dataset. All respondents for which this information was provided (n = 16; 89 % of the total sample of 18 women) were White. Half of the study sample had obtained a Master’s or doctoral degree, nearly half had some college education whether or not they obtained a degree, and one did not answer the question. Table 1 presents additional characteristics of the 18 key informants. The reference pregnancy was the first for 14 (78 %) respondents; the remaining four were in or had completed their second pregnancy. Outcomes of other pregnancies were not determined.

**Table 1:** Characteristics of 18 key informants

Status	Years from diagnosis to interview, *y*	Diagnosing clinician	No. of pregnancies^a^	Pregnancy intention for index	Type of vulvodynia
Pregnant	17	OB/GYN	1	Not trying to prevent	Secondary
Pregnant	10	OB/GYN	1	Trying	Secondary
Pregnant	1	Primary Doctor	1	Trying	Secondary
Pregnant	3	OB/GYN & Derm	2	Trying	Secondary
Pregnant	1	Uro-GYN	1	Trying	Secondary
Pregnant	5	OB/GYN	1	Trying	Secondary
Pregnant	5	OB/GYN	1	Trying	Primary
Pregnant	N/A	N/A	2	Trying	Primary
Postpartum	2	OB/GYN	2	Trying	Secondary
Postpartum	8	OB/GYN	1	Trying	Secondary
Postpartum	4	OB/GYN	1	Trying	Secondary
Postpartum	8	NP	1	Trying	Primary
Postpartum	6	OB/GYN	1	Trying	Secondary
Postpartum	4	OB/GYN	1	Trying	Primary
Postpartum	15	NP	1	Not trying	Secondary
Postpartum	4	OB/GYN	2	Trying	Primary
Postpartum	6	OB/GYN	1	Trying	Primary
Postpartum	8	OB/GYN	1	Trying	Secondary

^a^Including index pregnancy. *NP*, Nurse practitioner; *Derm*, Dermatologist

All but one respondent had been formally diagnosed with vulvodynia (any type) by a medical professional. The one respondent who had not received a diagnosis was eligible because she had symptoms consistent with vulvodynia. When asked during the interview to describe their pain, one third (6 of 18) described having symptoms from first vulvar contact with sex or tampon use, which is consistent with “primary vulvodynia”, while the remaining two thirds (12) described vulvar pain symptoms that onset after periods of pain-free sex and tampon use, consistent with “secondary vulvodynia”. The average time from diagnosis to the study interview was 6.3 years with a range of 1 to 17 years. Average age of respondents was 31.9 years with a range of 26 to 40 years. Six respondents were between the ages of 25 and 30, nine were between ages 30 and 35, and three were between 35 and 40. All participants were currently or previously in heterosexual relationships; 17 of the 18 (94 %) currently had a male partner. Sixteen of the 18 (89 %) respondents had actively sought pregnancy at the time they conceived.

We summarize in Table 2 the findings related to four themes that correspond more with the interview framework that focused on: 1 & 2) participants’ histories of pain and access to pain-focused health care, and subsequent attitudes, expectations and experiences related to said pain; 3) reproductive planning; and, 4) stages of conception, pregnancy, delivery, and, for those who delivered before the interview, postpartum.

**Table 2:** Summary of major themes and sub-themes

1. Pain Experience
a. Pain initiation
b. Pain volatility
c. Pain anxiety
d. Pain management
2. Interaction with Providers
3. Reproductive Decision Making
a. Pain minimization
b. Pain accommodation
4. Pain and Reproductive Milestones
a. Trying to conceive
b. Hope for improvement
c. Emotional reactions to pregnancy pain experience
d. Pain and delivery
e. Postpartum changes in pain

### Vulvar pain history

To contextualize pain in deciding whether or not to have a baby, it is important to consider a woman’s history of vulvar pain. During interviews with participants, we asked them to describe their pain history and significant events from the time they began to experience symptoms of vulvodynia to the present. Most gave detailed accounts of pain, including three emotional and cognitive states that were revealed in our analyses as key categories of this first major theme: 1) pain initiation or the point when a woman realizes her pain is not normal; 2) ongoing anxiety about her unpredictable pain severity, frequency, and duration; and, 3) pain management strategies developed over time through personal trial and error and/or medical intervention.

### Pain initiation

Several participants described a time when they were pain-free, which is consistent with secondary vulvodynia, before they experienced periodic *flare-ups* of chronic pain. Participants who reported always experiencing some level of pain consistent with primary vulvodynia came to a point in their lives when they realized their pain was not normal. For our study participants, the experience of pain was individualized and variable, a *moving target* in severity, frequency, and duration often with no identifiable triggers. One participant succinctly describes this variability in the following quote:

*“When I’m having a problem, it can last for several hours and be very, very severe and then disappear pretty much as quickly as it came. [After that,] I don’t have any problems for days, weeks or months.”* (Participant 1)

Similarly, another participant described her early history of pain severity and duration triggered by sexual intercourse with a hint of its change over time:

*“It seems like the burning pain with penetration that I had always experienced was initially something that was most intense within the first 30 s to minute of sex and then it would decrease. It would come back sometimes if sex was taking longer or if I didn’t have enough lubrication. Generally speaking, it was just in the beginning, enough that it almost takes your breath away, and then it would decrease.”* (Participant 10)

### Pain anxiety

Participants expressed nearly constant anxiety about their pain because onset seemed so unpredictable. One woman described in detail the emotional difficulties she faced with the situation:

*“I felt like I didn’t know how I was going to be from day-to-day. It wasn’t really that I couldn’t do things; but, for me, the anxiety of not knowing if I was going to be in pain while I did things was the worst part.”* (Participant 14)

While some of this anxiety stemmed from not knowing its origins, several women could describe exactly when and how their pain was provoked. More often than not, it was during sexual intercourse.

Several participants described hyper-vigilant cognizance of their anxiety and fears about pain during intercourse. As one woman put it, she has to be *“very careful”* during sex because she is *“a little afraid of that pain.”* (Participant 4) This was true whether or not the individual currently experienced day-to-day pain symptoms, as they often thought about the potential for triggering pain. One woman described her long term fears in this way:

*“I don’t know how to explain [it], other than having that excruciating pain for several months and then the moderate pain for several months, and then having pain from sex. I don’t know if I’ll ever be the same, in terms of having sex without fear of pain.”* (Participant 8)

Regardless of anxiety and fears, women in our study voiced a need to navigate through it, especially if the pain worsened due to pregnancy.

### Pain management

In response to their pain symptoms, anxiety, and fears, several participants independently sought information about vulvar pain and, armed with that knowledge and awareness, they developed ways of coping with, managing, accepting, blocking, or avoiding pain, including seeking medical guidance, which will be covered in the next major thematic section. Even though their pain had not gone away, these participants talked about improvements in their day-to-day lives using these strategies.

*“I always have pain with intercourse. In fact, even just manual stimulation or oral sex can be a little bit painful, too; so, I always have some level of pain that I try to just block out.”* (Participant 12)

For those participants who had personal pain management strategies, their approaches fell into four categories: clothing, products, physical and cognitive actions. Several women found they were more comfortable wearing skirts, dresses, and, loose-fitting pants and underwear. Others removed all harsh soap and fragrance products that irritated their skin and used, when pain got really bad, over-the-counter products such as lidocaine gel and other numbing or calming lotions and creams. Some found physical actions helpful such as lying down, engaging in physical therapy, or focused attention on muscle-relaxing exercise, or, they avoided sexual intercourse altogether. Still others shifted their cognitive perspective by trying to keep a positive attitude, and dealing with or blocking out the pain. While they used personal pain management strategies with no particular consistency, these participants expressed comfort in knowing they could use one or more of them when they needed it. A woman who never sought medical treatment for her pain said:

*“I’m married. [The pain] doesn’t prevent me from having sex. I can still have an orgasm. What I’ve looked into for [medical] treatment options…I haven’t felt like it’s been needed yet or totally necessary.”* (Participant 10)

Several study participants felt better equipped to cope with their pain simply because it had lessened or improved over time. One individual described this improvement like this:

*“I’m not saying it’s gone away; but, in a sense, I think it has improved. I used to tear up and cry during a vaginal exam at the doctor; and, at least now, I could tolerate that a little more.”* (Participant 9)

Individuals who experienced this type of pain improvement often had a hard time pin-pointing a specific explanation for it. One woman’s insight is telling in this regard:

*“I can’t say that any doctor or treatment really helped me; I think it was just [better] over time.”* (Participant 14)

When speaking of their experiences, our study participants described their reaction to pain as volatile at first, and, over time, more self-controlled as they moved away from medical treatments and healthcare systems, in general. While this does not mean that the pain completely subsided, once the volatility became more stable and overall severity lessened, many women in the study began planning for and attempting to achieve pregnancy. Before we move to the reproductive planning theme, we cover next our participants’ history of dealing with vulvar pain inside the health care system.

### History of navigating health care systems

When discussing the connection between vulvodynia and pregnancy, it is important to understand women’s experience with health care providers because of its influence on perceptions and expectations of health care before, during, and after pregnancy. We summarize useful highlights from current studies before describing our study findings.

Knowledge and familiarity with vulvodynia and its implications varies greatly by individual medical provider and across health care systems. Studies have shown patient frustration with provider mistreatment, insensitivity, disrespect, or dismissal of their condition [12, 18]. Women are often told that there is nothing wrong with them when physical indicators of disease could not be found [6]. Connor’s research team points out that women are required to be strong self-advocates when seeking diagnosis and treatment because most providers lack knowledge of effective treatments for vulvar pain disorders [12]. In a 2013 study, Nguyen and her colleagues found that women who sought care for vulvar pain were more likely to feel stigmatized potentially because of their evident frustration with the process of seeking a medical diagnosis and treatment [7]. Finally, due to the paucity of providers that specialize in vulvar pain, many women who experience these problems travel long distances to find a knowledgeable, experienced, and qualified practitioner [12].

In our current study, participating women diagnosed with vulvodynia validated findings from prior research. For instance, they often spoke of having to be their own experts and advocates and of their frustration in seeking qualified help. While some participants had neutral or positive experiences with health care providers, others talked about their exhausting and disheartening experiences through the process of explaining symptoms to physicians who were not aware of the disorder. Regardless of the degree to which these experiences were positive, most participants developed mistrustful attitudes toward future dealings with health care systems and providers. For example, one woman felt she:

*“…could have avoided this [vulvodynia] if I’d been smarter. It made me not trust doctors because I don’t think they necessarily know anything and I think they usually take the easy way out.”* (Participant 18)

Our participants also reported instances of doctors disregarding their complaints, sending them away with no further treatment options, or making them feel like they were *crazy*, as illustrated in this quote*:*

*“I guess my biggest frustration was just how dismissive doctors were. [They] would literally say that it’s probably just in your head. Maybe you should see a sex therapist.”* (Participant 6)

Another participant summed it up this way: *“…the [doctors] make you feel like you’re either A, crazy, or B, overreacting.”* (Participant 11)

Other barriers to receiving quality health care are inconsistent access to care across health care systems and among physicians. Many participants noticed their first symptoms during their college years or when they began post-education careers. For many young women, these life events often bring geographic relocation and transfers to new health care systems. In cases of relocations from family homes to distant colleges, participants described seeing a host of non-specialist providers such as family doctors during school breaks, and, at college clinics, ever-changing collections of general medicine physicians, nurses, and student practitioners in training. This fragmented care can make an already complicated chronic pain treatment course even more difficult to administer and follow successfully. Of those women who had seen a vulvodynia specialist for treatment, most were seen for a brief period during a flare up or to get a diagnosis. Few said during the interview that they still saw the doctor who diagnosed their disorder.

Additional barriers mentioned by participants to working with specialists included the high cost of specialized care and treatment, lack of coverage for such care by most insurance companies, and a scarcity of specialists to cover the volume and geographic dispersion of need for their services. When a specialist is located, long waits for appointments and the remissive and recurrent nature of the disorder also complicated access to medical help for study participants.

Given these experiences, we asked participants for suggestions to improve interactions with health care providers who are likely to see potential vulvodynia patients in their clinic setting. Several women talked about the importance of empathetic listening and strategies to alleviate patient fears and lessen anxiety about the diagnosis, symptoms, and isolation of vulvodynia. Several proposed open communication between patient and provider nurtured by mutual recognition of the emotional aspects of pain. One woman described it this way:

*“I think that the psychological component is so strong with [vulvar pain], or the anxiety component is so strong, that any bit of encouragement helps.”* (Participant 8)

Another participant approached the emotional aspect from this angle:

*“I think at that stage in my life I was really embarrassed by the pain, too. I wasn’t married and I didn’t want to talk about being sexually active and it being so painful.”* (Participant 9)

Some participants recommended changes at systems levels such as standardized training on vulvodynia at medical schools, vulvodynia screening as part of standard gynecologic practice, and training on effective patient referrals to providers who are unfamiliar with vulvodynia symptoms and treatment options. These systems-level recommendations align with those advanced by Rosenbaum and Padoa who advocated in 2012 for a coordinated care model for women with vulvar pain [13].

### Reproductive planning

Our primary research goal was to learn about the experiences of women who plan to have children and how vulvodynia symptoms and prognosis affect those plans. What follows is a summary of our findings on the influence of historical experiences with pain and ways our medical system affects the planning process. In the next section, subsequent expectations and reality of vulvar pain during conception, pregnancy, delivery, and postpartum are summarized.

All but two study participants described actively trying to get pregnant at the time they got pregnant. When asked about their decision to have a baby, most said their primary reason was timing. For example, the woman and her partner wanted a child, the woman’s partner wanted a child, or the woman and/or couple felt a need to start or increase family size because they could not “*wait much longer*.” “*I am 32, almost 33*,” said one participant, adding, *“If we’re going to have kids, we gotta do it now.”* (Participant 7)

Secondary factors related, more or less, to vulvar pain and are categorized here as pain minimization, timing of remission and treatment timing, and infertility.

### Pain minimization

Some participants pointed out that they would wait to plan a pregnancy if their current pain was worse. They often seemed to minimize their pain, as though it is not bad enough to warrant delaying pregnancy. One woman, who recently delivered her second child, seemed to minimize her vulvar pain when she answered the interviewer’s question about how vulvar pain affected her decisions to have a baby:

*“Maybe not at all. The reason we had him was because we wanted two kids to be close so they could play. Otherwise we would have maybe waited longer. We were just hoping to have our kids…they’re under two years apart, that’s what we were hoping for. Again, if it had been terrible and traumatic, we would have waited and gotten more treatments; but, it was just at a bothersome level at that point. It didn’t affect our decision to have another baby.”* (Participant 16)

Related to minimizing pain, the respondent went on to explain her acceptance of her vulvar pain:

*“It’s not like you’re going to wake up and [the pain’s] gone. ‘Okay, now I can have a baby.’ You have to go through it and have your children that you wanted to have anyway. If it [were] going to cause a further medical problem, then I would rethink it. At this point, I’ve just accepted it.”* (Participant 16)

### Pain remission timing

Some respondents also talked about how, though not gone, their pain had improved to a point where they felt comfortable getting pregnant. One woman described her experience with the pain before it improved as:

*“When I was burning three weeks out of the month and only not burning the week of my period, there was no question I was not going to get pregnant. I was not going to do it to myself.”* (Participant 7)

In this particular instance, the individual’s pain symptoms improved somewhat before she was ready to start trying to become pregnant.

### Pain treatment timing

A few participants were taking prescribed pain medication that, by doctor’s order, they discontinued in anticipation of being pregnant; they reported few or no problems using that strategy. Others felt they would delay pregnancy if they found an effective pain treatment regimen and needed more time with that treatment before getting pregnant. Several said that, without an effective treatment, they would have waited to get pregnant if their pain had been worse. Treatment to reduce or cure vulvar pain for these participants was clearly an important factor for reproductive planning, as this quote illustrates:

*“Honestly, if I had gotten treatment in the throes of it…when it was very severe, and [the medicine] had worked and someone told me you can’t have kids on this medicine, I think I would have just stayed on the medicine and not gotten pregnant again because that’s how severe it was…I probably would have been really grateful to have that pain go away.”* (Participant 1)

Another participant spoke explicitly about the importance of her pain treatment:

*“We were ready about a year prior, but I wanted to continue with the physical therapy and hoped that I would have continued improvement… After delaying for about a year and having done physical therapy for about two years, we decided to just go ahead and go for it.”* (Participant 13)

### Infertility

Few women in the study mentioned experiencing infertility. Of those for whom infertility was an issue, all of these participants used some form of assisted reproductive technologies (ART) for that purpose. One woman who described her process of going through ART on top of her vulvodynia symptoms said this about her experience:

*“I think infertility for any couple is going to be emotionally trying and I think vaginal pain on top of that can really…I don’t know, it was a little bit more emotionally painful for me.”* (Participant 6)

Two women in our study used ART ovulation sticks, not for infertility, but for family planning in a way that carefully considered vulvodynia pain through strategically timed sexual intercourse. One of these women described the strategy in this quote:

*“We talked to the obstetrician who knows a lot about vulvodynia and he had suggested, since intercourse was so painful, that we do it in a time-conscious manner and use the ovulation sticks that you urinate on. Since it was painful for me, he said ‘It would be best if when you do ‘do’ it, you do it on the appropriate days.’”* (Participant 9)

### Pain in pregnancy: outcomes

To describe our findings on vulvar pain and pregnancy, we examine here study participants’ reports of pain during progressive stages from trying to conceive, to being pregnant, going through delivery, and into early postpartum for those in our study who had delivered by the time of the interview.

### Trying to conceive

Most participants were not in full remission from their symptoms at the times they tried to get pregnant or when they conceived. While many were anxious about conceiving, several described ways they tapped into pain management strategies such as avoidance, as this woman describes:

*“I wouldn’t say that we weren’t having sex because of it [the pain]. I would say we were having less sex because of it.”* (Participant 14)

Another participant’s experience seemed more difficult:

*“It was a lot harder for me because to have sex was so excruciating I would cry. It was just frustrating and frustrating to see other people that could just get pregnant right away…We were trying to limit the amount of time or the frequency, so that the irritation and burning wouldn’t be so bad.”* (Participant 9)

Prior experiences with pain and doctors combined with expectations about going through pregnancy also increased conception anxiety related to infertility and speculum exams. This participant spoke for others like her:

*“I would say probably a lot of women who have vestibulitis and were trying to get pregnant…it’s probably just slightly more emotionally trying for them just because every time they have sex, [it’s] painful.”* (Participant 6)

### Pregnancy pain

As noted, all of our participants were pregnant or had delivered one or more children at the time of the study interview. When they discussed pregnancy and pain experiences, their responses fell into three categories: 1) heightened anxiety for nearly everyone interviewed; 2) hope for symptom improvement based on several sources, such as friends, others with vulvar pain, and potentially health care providers; 3) emotional reactions to their various pain realities.

### Hope for improvement

Several participants heard from physicians and others or read that pregnancy could improve or *cure* their vulvodynia symptoms. One woman decided to get pregnant because “*I haven’t had a ton of symptoms”* and she *“had this hope, like well, maybe if I get pregnant … that maybe it wouldn’t be as bad.”* (Participant 7) For another participant, her doctor’s assertion that “*once I got pregnant … my symptoms would clear up a lot”* made a *“huge”* difference to her because *“I just wanted to have some pain-free days.”* (Participant 2)

### Emotional reactions to pregnancy pain experience

While no one in our study pursued pregnancy solely to improve pain symptoms, many participants clearly hoped to experience relief from their symptoms. This quote reflects an unspoken hope and open disappointment that reality would have been different for her:

*“I did really well for a long time and then I got pregnant [and tried to have sex and] it was terrible. It just hurt like it hadn’t hurt in years and it was just terrible. Pain like I had not felt since before the surgery. It was really disappointing.”* (Participant 5)

For a few women, disappointment related to old feelings of inadequacy or abnormality related to their condition. They talked about “*not [being] a real woman*” and not being capable of “*hav[ing] sex or do[ing] womanly sexual activities.*” (Participant 5) When she experienced this old feeling during her pregnancy, she described her emotional reaction like this:

*“I had just read in…‘What to Expect When You’re Expecting’…that sex was going to be really good while you’re pregnant and so I was looking forward to that; and, then when it didn’t happen, I just sort of felt again, like ‘oh, you know, something else is wrong with me.’”* (Participant 5)

Those who experienced a sudden increase in pain were disheartened and fearful that it would stay or get worse. It was especially difficult for those who had stopped successful pain treatment before conceiving. For them, the return to a pain state they had experienced at an earlier point in their chronic pain history was especially devastating and some feared they could not start *“dealing with it”* (Participant 11) if the pain was, in fact, getting worse. This participant’s plea for answers reflects an unfortunate absence of information for these women:

*“I [was] wondering, like, ‘oh my gosh…is this how it’s going to be after we give birth?… Are we going to have to start all over?”* (Participant 5)

Another participant described her emotional frustration with the lack of answers this way:

*“[You’re] being told by doctors ‘unfortunately, you have to go through your pregnancy and have the baby and then wait in order to find out if it has returned.’ It’s very frustrating because you don’t want to wait. You don’t want to wait another year. You want to know now. You want to know if it’s back so that you can cope with it. Or, if it’s not back. Or, exactly what’s going on with your body? And, that’s…frustrating.”* (Participant 4)

In a few cases, participants found welcomed symptom relief during pregnancy. One woman described her anxiety with intercourse during pregnancy because she was “*afraid it would hurt … I was tensing up.*” (Participant 4) As her *“hormone levels increased and my lubrication increased”* her fear decreased and her pain got:

*“…better as the pregnancy progressed… [B]y the second trimester, things got so much better…the tissue was not sensitive at all.”* (Participant 4)

### Pain and delivery

Whether they experienced symptom improvement, stability, or worsening during pregnancy, almost all study participants were concerned about vulvar pain associated with child delivery. While pain anxiety regarding childbirth is common [19], our participants described a variety of reasons why their situation was unique. Having developed day-to-day pain management strategies prior to reproductive planning, several participants actively engaged in adjusting their birth plan to minimize or avoid pain. Some plans looked like these two:

*“I’m going to request a Cesarean because, if I understand correctly, this condition can worsen through vaginal birth. I don’t need it any worse than what it already is.”* (Participant 15)

*“We’re going to start with an epidural and then see how that goes from there. I’m not going to try natural, which is too bad because I’m a natural-type person; but there’s no way. If I can’t tolerate a penis, I don’t want to even think about anything else.”* (Participant 12)

Other approaches evolved over time from initial concerns and anxiety to anticipated resilience. Some participants adopted an attitude of acceptance about childbirth pain, which brought some degree of comfort for a few. Acceptance could be interpreted as minimization of pain or normalizing, and the next two quotes speak to this plausible interpretation:

*“Honestly, I think, you know, when I’m giving birth, I’m going to be in enough other pain that I’m not going to care what’s happening down there, you know?…I’ll deal with whatever it is…if I even notice if it’s happening.”* (Participant 3)

*“I was really concerned with how that would mesh with my condition; and, you know, they assured me that childbirth is pretty equally painful for just about everybody [chuckle].”* (Participant 6)

As with the hopeful wait-and-see approach to anticipating pregnancy pain, some pregnant participants hoped that a vaginal birth would help their symptoms. One participant who was in the postpartum stage at the time of the interview verified that relief from pain could be a reality for some:

*“I swear by that vaginal birth. I think it really helped [my symptoms]. You don’t realize that you’re clenching or you don’t realize that your muscles are tightening. Over a few years of vulvar pain, that area gets tight and that forced stretch of getting a nine-pound baby through there seems to really help. It hurt like crazy when the baby was [coming out], but I would do it all over again.”* (Participant 8)

### Postpartum pain

While pain or the perception of it may increase or lessen during pregnancy, delivery, and postpartum vulvar pain also appears to be as variable and individualized as it is at all other stages from pain initiation to childbirth and beyond. For one participant in her postpartum stage, the disappointment in persistent vulvar pain comes through in this quote:

*“My husband and I have had intercourse since I delivered. It doesn’t seem like it’s any worse than what it was prior to the baby and the delivery. I was hoping for a miracle; but, it looks like I’ll have to keep working on it.”* (Participant 13)

### Potential study limitations

This qualitative study was pursued to explore an area of reproductive health that is currently understudied and little known to researchers and health care providers. As with all such explorations, the study has limitations. We included only women who were pregnant or recently delivered a baby; therefore, we narrowed our understanding of the full spectrum of experiences a woman can have when considering pregnancy with vulvodynia. Our participants had different vulvar pain characteristics, for example, some women have always had pain with intercourse, while for others it was a newer experience. It could be speculated that the length of time with vulvar pain may affect a woman feels about reproduction. Yet, it is not known how these pain characteristics, as well other pain characteristics, may influence our summaries. At the onset, we did not specifically probe issues related to the women’s partners such as levels of encouragement and support, however, no theme about partners was identified by the participants. As with all qualitative studies, our research conclusions are situational as opposed to demographically representative. With this in mind, transferability of these findings to other groups experiencing similar situations should be encouraged with larger studies examining these themes.

## Conclusions

We have identified major themes that if addressed successfully, could reduce the burden of vulvodynia on women during their reproductive years. First, pain-related experiences prior to pregnancy strongly informed decision making during pregnancy. Women seemed to reach a personally acceptable level of pain before planning and achieving their pregnancy; we did not find that women were completely free of vulvar pain when deciding to become pregnant. Second, this study has identified relevant sources of frustration for pregnant women seeking care for both themselves and their pregnancy. Women commonly referred to a disconnect between gynecologic care for their vulvar pain and their obstetric care during their pregnancy. Respondents suggested that this gap in medical care should be addressed with changes in screening policy and increased medical education on vulvodynia. However, other age-specific factors influenced this disconnect as well, such as the transient nature of young adulthood that may affect a woman’s care continuity. Efficacious models of addressing and treating pain should be adapted to specifically address the conception-pregnancy-postpartum time period for these women. Finally, women in our study expressed many normative feelings of anxiety and hopefulness regarding pregnancy, which often times were representative of their resilience. Therefore, building resilience with the assistance of therapists, partners, and other individuals who provide social support to the women should be a primary focus of future research.
